# Supplementary material for: Physiological and Transcriptomic Analyses of Escherichia coli Serotype O157:H7 in Response to Rhamnolipid Treatment
Source: Microorganisms. 2023 Aug 18;11(8):2112. doi: 10.3390/microorganisms11082112 (PMC10459150; doi:10.3390/microorganisms11082112)
Supplement: Supplementary file 1 [file microorganisms-11-02112-s001.zip › microorganisms-2524284-supplementary.pdf]

Table S1. Primers used in the study of gene expression in *E. coli* O157:H7.

Gene name	Primer	Primer sequence (5′-3′)
lpxC	*lpxC*-F	ATTCCAGCAACCAGCGCTAT
	lpxC-R	CGATGGCACAATCGAAGCTG
lpxH	*lpxH*-F	GCGAACAGCAAAGAAGCCAA
	lpxH-R	GATAAGTTCATGCACCGCCG
fabA	*fabA*-F	GCAATTGCCAGCACCGAAC
	fabB-R	CGCGCGGCCTTTACCTTC
accA	*accA*-F	TTGCGCAACTGGCACGCCAT
	accA-R	GGCGCTGGCATACCAAAGTT
cyoA	*cyoA*-F	CGGCACTTATGACGGTATCTC
	cyoA-R	TCCACCTGGTTGTATTCGCTA
cyoC	*cyoC*-F	GATGGCGGTGCTGATGGT
	cyoC-R	CACATCCAGGAAGTGCCAGA
flgC	*flgC*-F	CGTTGATGTTGTCGGAGAGAT
	flgC-R	CGTAAGGGTTTTCAGCATCAT
flgD	*flgD*-F	TCTGACTTTGTTGGTGGCG
	flgD-R	CGACTGGCTGTTATCAATCTGT
pgaA	*pgaA*-F	CGTCGCTTATCGTAACCTGC
	pgaA-R	CGCAGTATCATAGTGACCAGCA
pgaB	*pgaB*-F	TGTTCCACGATGATGCTTTG
	pgaB-R	TCGAATTTCGCTCAGACTCC
dnaE	*dnaE*-F	ATGTCGGAGGCGTAAGGCT
	dnaE-R	TCCAGGGCGTCAGTAAACAA

Table S2. KEGG enriched pathways of DEGs treated with RL.

KEGG ID	Pathway	Gene name	Count	*p*-adj
**Up-regulated pathways**				
eco03010	Ribosome	*rpsJ/rplC/rplW/rplD/rplI/rpsS/rplB/rpsF/rpsC/rplV/ykgO/rplF/rpsE/rplO/rpsH/rpmD/rpsR/rpmC/rpsQ/rplK/rpsN/rplP/rplA/rplR/rplJ/rplE/rpsM/rplQ/rpsI/rpsK/rplN/rplL/rpsD/rpsB/rplM/rplX/rpsA/rpsG/rplS/rpmJ/rpmH/rpmB/rplU/rplY/rpmA/rpsT/rpsO/rpsL/rpsU/rplT/rpmI/rpsP/rpmF/rpmG/rpmE*	55	2.31×10^{-32}
eco00540	Lipopolysaccharide biosynthesis	*lpxP/lpxK/waaA/lpxB/eptB/lpxM/arnT/eptA/waaG/rfaQ/kdsA/lpxH/lpxD/lpxA/eptC/rfaC/rfaP/waaO/lpxC/lpxT/gmhB/rfaF/kdsC/rfaD*	24	1.1×10^{-4}
eco03070	Bacterial secretion system	*secY/yidC/secE/secG/secF/secA/secD/ffh/yajC/ftsY/gspK/secM/tatC/tatB/gspJ/tatE/tolC/gspL/escC*	19	4.5×10^{-4}
eco03440	Homologous recombination	*priB/dnaN/recO/dnaE/priA/ssb1/dnaX/holD/recR/ruvC/recB/recA/holA/recF/recG/recJ/dnaT/holB/ruvB/recD*	20	4.5×10^{-4}
eco00541	O-Antigen nucleotide sugar biosynthesis	*cpsG/ugd/galF/gmd/wecC/wecB/rffG/rffC/rfbA/rffA/galU*	14	4.5×10^{-4}
eco03060	Protein export	*secY/yidC/secE/secG/secF/secA/secD/ffh/yajC/ftsY/secM/tatC/lepB/tatB/tatE*	15	6.0×10^{-4}
eco00970	Aminoacyl-tRNA biosynthesis	*thrS/metG/glyS/hisS/aspS/lysS/glyQ/argS/cysS/proS/pheS/leuS/alaS/gltX/asnS/pheT/fmt*	17	0.01
eco03018	RNA degradation	*deaD/pnp/rhlE/rnr/rho/pcnB/recQ/rne/rhlB/eno*	10	0.02
eco00061	Fatty acid biosynthesis	*fabH/fabA/accC/fabD/ fadD/accA/accD/accB*	8	0.02
eco00670	One carbon pool by folate	*purN/purH/glyA/folD/thyA/purU/fau/folA/gcvT/fmt*	10	0.02
eco00190	Oxidative phosphorylation	*yoA/cyoB/nuoN/sdhC/nuoM/cyoC/cyoE/ppa/cyoD/nuoL/nuoJ/atpH/atpB/atpC/atpG/atpF/atpA/atpD/sdhD/atpE/nuoI/nuoH/nuoK/nuoG*	24	0.04
**Down-regulated pathways**				
eco01120	Microbial metabolism in diverse environments	*sseA/napA/fucO/ldhA/pflB/frdB/frdA/hybO/frdC/narG/mgsA/hchA/agp/aldB/pdxI/kdgK/gapA/frdD/tdcE/fbaB/cysQ/nifJ/yeaD/fumB/pyk/lysA/fumC/fucA/adhE/allC/allD/ulaC/ulaE/hybC/pta/narH/tkt/ackA/torA/ulaA/lysC/yihX/hemL/tkt/ydiF/fumB/torZ/rhaB/pfkB/torD/allB/tal/glk/*	93	2.13×10^{-8}

		ulaF/ulaD/narI/patD/xdhA/cadA/dmpG/maeB/narZ/glxK/ulaG/ahr/nrfA/yggF/ulaB/nfsA/csiD/hcaE/mhpD/eutG/ltaE/yihR/hyaA/cysN/gpmM/ybhJ/cysC/mhpB/galM/aceF/ppsA/thrB/narI/gabD/hcaD/eutE/asd/mhpC/hyaB/rhmD		
eco00040	Pentose and glucuronate interconversions	*uxuB/uxuA/uxaC/araA/uxaB/xylA/uxaA/fucA/ulaE/araB/xylB/rhaB/kduI/ulaF/ulaD/kduD*	16	2.2×10^{-4}
eco00620	Pyruvate metabolism	*ldhA/pflB/frdB/frdA/frdC/hchA/aldB/frdD/tdcE/nifJ/fumB/pyk/fumC/adhE/pta/ackA/fumB/pta/poxB/maeB/ahr/eutG/aceF/ppsA/maeA/eutE*	26	3.0×10^{-3}
eco00053	Ascorbate and aldarate metabolism	*garD/ulaC/ulaE/ulaA/gudD/ulaF/ulaD/lgoD/ulaG/ulaB*	10	3.0×10^{-3}
eco00052	Galactose metabolism	*agaV/kbaZ/melA/galT/pfkB/glk/agaI/galK/gatZ/yihR/gatY/galM*	12	3.0×10^{-3}
eco00010	Glycolysis / Gluconeogenesis	*malX/agp/aldB/gapA/fbaB/nifJ/yeaD/pyk/adhE/yihX/pfkB/glk/ascF/ahr/yggF/eutG/yihR/ascB/gpmM/galM/aceF/ppsA*	22	3.0×10^{-3}
eco02060	Phosphotransferase system (PTS)	*malX/mtlA/cmtA/agaV/ulaC/murP/treB/ulaA/nagE/ascF/ulaB/srlE/cmtB*	13	0.01
eco00650	Butanoate metabolism	*pflB/frdB/frdA/frdC/frdD/tdcE/nifJ/adhE/ydiF/ilvM/yihU/dmlA/ilvN/gabD*	14	0.01
eco00051	Fructose and mannose metabolism	*mtlA/fbaB/mak/xylA/cmtA/fucA/rhaB/pfkB/mtlD/sorE/hxpA/yggF/srlE/cmtB/rhmD*	15	0.05
